# Allel RET+3 : T a medullary thyroid cancer

**DOI:** 10.1186/1897-4287-9-S2-A12

**Published:** 2011-06-01

**Authors:** A Pławski, M Podralska

**Affiliations:** 1 Institute of Human Genetics, Polish Academy of Sciences, Poznan, Poland

## 

In our studies, we compared the frequency of the occurrence of the RET+3 : T allele in our group of 48 medullary thyroid cancer (MTC)patients with the frequency of occurrence of the allele in the Polish population. The frequency of the occurrence of the heterozygote variant of the RET+3 : T for the Polish population reached almost 12% (18/152) of heterozygotes, but in the group of patients with MTC, we did not find even a single RET+3 : T allele. The frequency difference is statistically significant and in the Fisher's Exact Test, the two-sided P value is 0.0080. This observation allows assuming that the occurrence of the RET+3 : T in the heterozygotic state may lead to the inhibition of the disease phenotype in the cases of the medullary thyroid carcinoma.

